# High prevalence of *Paenibacillus larvae*, the pathogenic agent of American foulbrood disease, in Palestinian honey bee colonies

**DOI:** 10.1007/s11259-025-11034-x

**Published:** 2026-01-19

**Authors:** Mohammad Alqurneh, Nino Tuan Phong Bergmann, Islam Nairoukh, Abdul-Jalil Hamdan, Hans-Hinrich Kaatz

**Affiliations:** 1 Institute of Biology, Zoology, Martin-Luther-University Halle-Wittenberg, Saxony-Anhalt, Germany; 2 Department of Pest Monitoring and Control, General Directorate of Plant Protection and Inspection Services, Ministry of Agriculture, Ramallah, Palestine; 3 Department of Plant Protection and Production, Agriculture Science College, Hebron University, Hebron, Palestine

**Keywords:** Apis mellifera, American foulbrood, ERIC genotypes, Honey bee, Paenibacillus larvae

## Abstract

The spore-forming bacterium *Paenibacillus larvae* causes a devastating brood disease of honey bees (*Apis mellifera*) named American foulbrood (AFB). It occurs worldwide and can lead to the death of whole colonies. Presence and prevalence of this pathogen in Palestine were unknown. Therefore, for two consecutive years, a field survey was conducted to screen Palestinian beekeeping locations for *P. larvae*. Honey samples were collected from 10 colonies each of eight apiaries throughout the West Bank in spring, summer, and fall. The presence of the bacterium was identified microbiologically, via PCR and DNA sequencing. Bacterial genotypes were analysed by repetitive element-PCR fingerprinting using ERIC primers. We unexpectedly detected spores in 87.5% of the inspected apiaries, 22.3% of 130 inspected colonies contained spores. 62.1% of the colonies with spores were lost during the study period. Whereas clinical symptoms of the disease were absent, parameters like reduced brood production and or spotty brood pattern proved to be helpful but indirect indicators for the disease. The detection of *P. larvae* is the first record of this fatal pathogen in Palestine. The genotype ERIC I was found six apiaries, the more virulent ERIC II genotype was present in two apiaries and is the first report of ERIC genotypes in Palestine. The study emphasizes the need for immediate steps toward efficient treatment limiting the spread of this harmful bacterium within the country and in neighboring countries.

**Supplementary Information:**

The online version contains supplementary material available at 10.1007/s11259-025-11034-x.

## Introduction

The honey bee (*Apis mellifera* L.) is one of the most valuable pollinators for many crops, wild flowering species, and the fruit industry (Klein et al. 2007). The demand for insect pollination for farming has tripled during the last 50 years. However, the number of managed honey bee colonies decreased in many regions of the world as USA, the Middle East, and Europe by 30%, 10–85%, and 1.8–53%, respectively (Neumann et al. 2010; EPILOBEE Consortium et al. 2016). Honey bee colony losses have also been reported for South America (Maggi et al. 2016) and Africa (Nganso et al. 2025). These colony losses were driven by several factors including parasites like the mite *Varroa destructor*; diseases like American Foulbrood or virus infections; beekeeping practices, changes in climatic conditions; agricultural practices, and the use of pesticides (vanEngelsdorp and Meixner 2010; Smith et al. 2013; Lin et al. 2023).

Among the biotic drivers of colony losses, American foulbrood (AFB) disease affecting honey bee brood is one of the most significant threats, leading to the death of entire honey bee colonies (Genersch 2010; Poppinga and Genersch 2015; EPILOBEE Consortium et al. 2016; Morawetz et al. 2019). AFB is caused by the gram-positive, rod-shaped, spore-forming bacterium *Paenibacillus larvae (P. larvae)*. Only the spores infect the larval stage of honey bees. A dose of about ten or fewer spores is able to infect the larvae. The optimal period to infect larvae is 12–36 h after egg hatching (Genersch et al. 2006). The life cycle of *P. larvae*, reviewed by Genersch (2010), starts with the germination of spores in the midgut lumen, then the vegetative cells massively proliferate before they penetrate the larval midgut epithelium and invade the haemocoel. The bacteria sporulate in decaying larvae, and the remains disintegrate into a ropy mass. The dead larva, prepupa or pupa may contain as much as 2.5 × 10^9^ spores (Lindström et al. 2008a). These spores are infectious for more than 35 years (Hasemann 1961).

The ropy mass is a typical clinical symptom for AFB that can be identified with a matchstick during the inspection (Genersch et al. 2006). Additionally, there are other specific visual clinical symptoms of AFB infection, including dead larval remains that become a tough but brittle scale, which is difficult to remove from the cell, brown dead pupae with tongues stretching across brood cells, and a slightly foul odor. Unspecific indicators of AFB infection comprise sunken, dark and perforated wax cappings containing brownish larvae or a spotty brood pattern, a scattered arrangement and irregularly patterned brood area appearance with a mixture of empty, open and capped cells (de Graaf et al. 2006; Genersch 2010; OIE, 2018). The empty cells in the spotty brood area appear after early age larvae were killed by *P. larvae* and removed by adult workers (Brødsgaard et al. 2000).

Control and combat against AFB are still the most challenging and complex operations due to the tenacious spores. Three major treatments are effective (Genersch 2010). First, burning colonies and contaminated hive material still are widely considered the only workable control measure for AFB diseased colonies. Second, the shook swarm method (shaking the bees onto a new comb foundation and destroying the infected combs) has been successfully applied to sanitize infection (Pernal et al. 2008). Third, antibiotics such as oxytetracycline hydrochloride (OTC), tylosin and lincomycin are used to treat infected colonies (Kochansky et al. 2001; Pettis and Feldlaufer 2005; Alippi et al. 2005). but they are banned in most European countries due to residues in the honey. Antibiotics effectively inhibit the growth of the vegetative form of bacteria but do not kill the long-living *P. larvae* spores. Thus, they affect the symptoms and do not cure but mask the disease.

Diagnosis of AFB is based on identifying the pathogenic agent in larvae or pupa and the existence of clinical symptoms. However, in practice, the type of sample collected depends on the situation—whether it involves a suspected or clinically affected colony/apiary, or routine testing as part of an AFB surveillance and prevention program (de Graaf et al. 2013; OIE, 2018). In several countries, quantifying *P. larvae* spores in honey stored adjacent to sealed brood is employed as an indicator for monitoring and assessing the risk of AFB outbreaks (Bassi et al. 2018).

Detection of *P. larvae* is based on classical microbiological identification methods from sources like larvae, adult bees, honey and hive debris (Forsgren and Laugen 2014; Bassi et al. 2018) and complemented by fast and precise molecular techniques – PCR, real-time PCR and sequencing. Specific PCR primers based on 16 S rRNA gene of *P. larvae* were designed and commonly used for molecular AFB detection (Govan et al. 1999; de Graaf et al. 2013; Carra et al. 2022; Okamoto et al. 2022). Biologically and phenotypically different *P. larvae* genotypes were determined using enterobacterial repetitive intergenic consensus (ERIC) sequences (Versalovic et al. 1994; Genersch et al. 2006). Five genotypes (ERIC I-V) of *P. larvae* had been identified (Genersch et al. 2006; Beims et al. 2020). ERIC I and ERIC II are the primary *Paenibacillus larvae* genotypes associated with clinical outbreaks of American foulbrood (AFB) in honey bee colonies (Genersch 2010) and frequently identified worldwide (OIE, 2018; Hristov et al. 2021). While other ERIC genotypes (III - V) have been isolated from honey and hive materials, their role in causing AFB remains unconfirmed (Genersch et al. 2006; Beims et al. 2020). The genotypes differ in speed of killing larvae: slow in ERIC I (LT_100_ 7–12 days), medium in ERIC II (LT_100_ 5–7 days), and fast in ERIC III, IV and V (LT_100_ 3 days) (Genersch et al. 2006; Beims et al. 2020). Hygienic bees recognize and remove infected brood from the nest. Slow-killing ERIC I genotypes generate typical clinical symptoms such as dead larval remains and infected pupa with tongues stretching across brood cells. In contrast, in ERIC II-V medium and fast killing variants, hygienic bees usually remove the infected larvae before cell capping resulting in empty brood cells (Brødsgaard et al. 2000) and in the absence of typical clinical symptoms but generating a scattered brood pattern. Thus, ERIC II genotypes may be less easily detected.

*P. larvae* is present in almost all beekeeping regions on all continents (OIE, 2009; Hristov et al. 2021), but its colony prevalence varies between 0.3 and 44,7% on a country-specific level (Arabiat et al. 2012; EPILOBEE Consortium et al. 2016; Genersch et al. 2010; Hall et al. 2021; Hristov et al. 2021; Hulaj et al. 2024; Alburaki et al. 2025). The knowledge about the occurrence of AFB in the Middle East and North Africa is limited (Arabiat et al. 2012; Hamdi et al. 2013; Ansari et al. 2017). The current study aims to determine the occurrence, prevalence, and genotypes of *P. larvae* in Palestine using microbiological and molecular identification methods and evaluate the impact of this disease on colony losses.

## Materials and methods

The research was conducted for two years (2017–2018). In order to identify the prevalence of *Paenibacillus larvae* in Palestine, eight apiaries of different beekeepers were selected from two model governorates in West Bank: Bethlehem and Hebron. The regions were selected as study regions due to their representative Mediterranean climate and central highland location, and significance in Palestine’s beekeeping sector, hosting large human populations and thousands of beehives, making them key centers for beekeeping activities in Palestine.

These apiaries were visited three times (spring (March), summer (July), and autumn (October) per year, and ten randomly chosen colonies per apiary were inspected at the beginning and monitored throughout the study. The visit schedule followed the German monitoring program and the pan-European EPILOBEE protocol (Genersch et al. 2010; EPILOBEE Consortium et al. 2016). If a colony collapsed in the course of the study, it was replaced with another randomly chosen colony of the same apiary to keep the total number of 80 monitored colonies consistent per sampling period. Colony losses were recorded relative to the previous visit, with autumn 2018 losses evaluated in spring 2019. Data on the symptoms and the bacterial loads at the time of colony collapse were not available since the colonies died in the unobserved intervals between the visits.

**Fig. 1:**
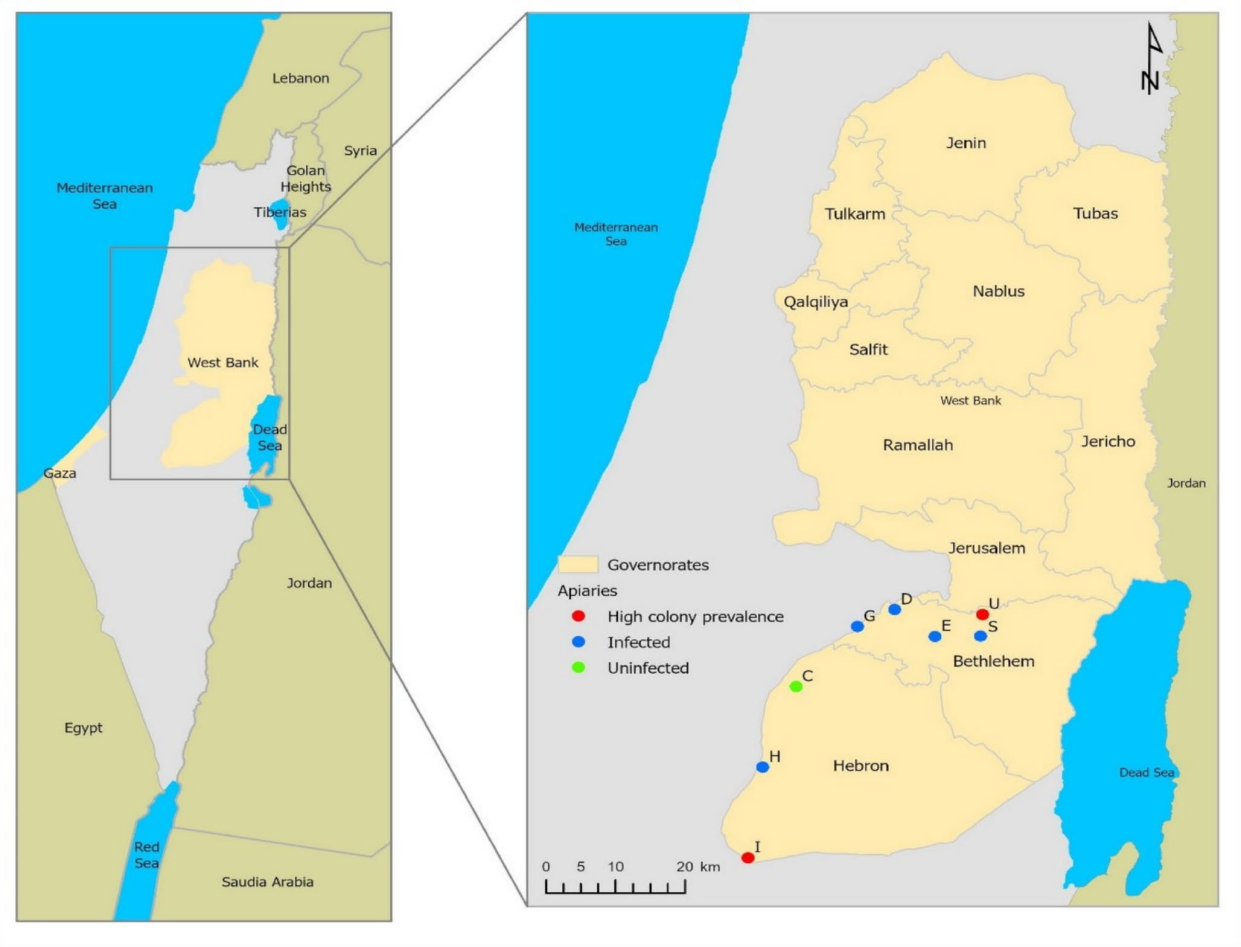
Location of the inspected apiaries in the West-Bank region of Palestine and the occurrence of *Paenibacillus larvae* spores. This pathogen was detected in seven out of eight apiaries (green dot: undetected, blue dot: detected in < 50% colonies per apiary, red dot: detected in > 50% colonies per apiary). Letters represent the following apiaries: Ubediya (U), Hindaza (E), Battir (D), Wadi Fukin (G), Shawawreh (S), Al-Dahiriya (I), Majd (H), and Bayt-Ula (C). Map resource: Geomolg Portal, a local web mapping application, was administrated by the Ministry of Local Government (see the statistical analysis section for more details)

The apiaries were located at the following geographical coordinates (Fig. 1): C. Bayt-Ula (31° 36’ 21.247"N, 34° 59’ 31.395"E), H. Majd-Dura (31° 29’ 8.762” N, 34° 56’ 31.179” E), I. Al-Dahiriya (31° 21’ 3.418” N, 34° 55’ 12.593” E), D. Battir (31° 43’ 14.217” N, 35° 8’ 31.826” E), E. Hindaza (31° 40’ 50.893” N, 35° 12’ 13.963” E), U. Ubediya (31° 42’ 48.422” N, 35° 16’ 33.185” E), G. Wadi-Fukin (31° 41’ 42.946” N, 35° 5’ 8.343” E), S. Shawawreh (31° 40’ 52.216” N, 35° 16’ 23.893” E).

### Data collection and sampling

In a questionnaire, all cooperating beekeepers were asked about occurrence of American Foulbrood disease and treatment regimes against *P. larvae* including type, chronological pattern and chemicals used. None of the beekeepers had seen symptoms of AFB but colonies of five apiaries were preventively and regularly treated against AFB with the antibiotic oxytetracycline hydrochloride (5 g dissolved in 1 L 50% sugar solution (w/v) per colony; cf. Tabel 1) once a year in early spring (February or March) before blooming season.

Honey bee colonies which represent hybrids of local (*Apis mellifera syriaca*) and imported Italian honey bees (*A. mellifera ligustica*) were visually inspected for any clinical symptoms of AFB disease during each inspection phase (Genersch 2010; OIE, 2018). Additionally, a honey sample (10–20 g) was collected from cells close to the brood of each colony (Ritter and Kiefer 1995; Antúnez et al. 2004; Bassi et al. 2018) in order to detect *P. larvae* spores and spore loads. In fall 2018 larval samples were collected for *P. larvae* identification from colonies with spotty brood patterns (OIE, 2018).

### Colony strength and assessment for capped brood pattern

During colony inspection, all sides of the frames were photographically documented with a CMOS digital camera with a macro lens. Colony strength (number of adult bees, number of open and capped brood cells) and food stores in wax-capped cells of the combs were estimated using the standard Liebefeld method (Delaplane et al. 2013). Photographs were additionally used for bee brood assessment conducted at frames with capped brood. The number of open cells within these areas indicate failures of normal brood development (Delaplane et al. 2013). Areas with capped brood cells from three random separate brood frames of a colony were selected and the proportion of open cells within 3 × 100 capped brood cells was counted. Comparisons between spore-containing and spore-free colonies were drawn at the endpoint of the study or one season before a colony died.

### Isolation and cultivation of *P. larvae* from honey samples

The respective honey samples were dissolved in the same amount (v/w) of H_2_O and heated to 90 °C for 6 min to kill vegetative cells. After cooling to room temperature, samples of 100 µL were plated in triplicates on MYPGP agar (Mueller-Hinton broth, yeast extract, pyruvic acid sodium salt, D (+)-Glucose anhydrous, agar bacteriology grade and di-potassium hydrogen phosphate) (de Graaf et al. 2013) and incubated for six days at 37 °C. After counting the number of colonies morphologically typical for *P. larvae* (small, regular, mostly rough, flat or raised and whitish to beige coloured) (Genersch et al. 2006; Beims et al. 2020) the number of viable spores was calculated and expressed as colony-forming units (CFU) per g of honey. A total of 356 honey samples were collected and analyzed for the presence of *P. larvae* spores.

### Molecular identification of *P. larvae* from bacterial isolates and larval samples

All bacterial colonies morphologically identified as *P. larvae* were subjected to molecular identification by PCR fragment analysis via a fast-screening method. Colonies were picked with a sterile pipet tip, suspended in 50 µl distilled water, heated at 100 °C for 6 minutes, and centrifuged at 10,000 rpm for 5 minutes. A 0.2 µl aliquot of the supernatant was analyzed by PCR using primers 1–5’- AAGTCGAGCGGACCTTGTGTTTC-3’; primer 2–5’- TCTATCTCAAAACCGGTCAGAGG-3’ (Govan et al. 1999). PCR reaction was performed at 10 µl volumes containing 1 µl 10x buffer, 0.2 µl dNTPs (10 mM), 0.05 µl Taq-polymerase (5 U, Promega, Mannheim, Germany), 0.2 µM of each forward and reverse primer and 0.2 µl of DNA template, 8.15 µl of d.d.H_2_O. PCR parameters for amplification were: one cycle at 95 °C for one min, followed by 35 cycles at 93 °C for one min, 55 °C for 45 s and 72 °C for one min and a final extension cycle at 72 °C for 5 min. The fragment size of the PCR product was either determined by capillary gel electrophoresis (QIAxcel, Qiagen GmbH, Germany) or by agarose gel electrophoresis (1% agarose, TBE-buffer). The approximate product size of the latter, Midori Green (Nippon Genetics, Japan) stained amplicons were determined using the 100-bp molecular size marker (Promega, USA). The amplicons were visualized and photographed using a BioDoc Gel Analyzer (Biometra, Germany). The bacterial DNA extracts were stored at −20 °C for further analysis.

Due to the absence of typical clinical AFB symptoms, larval samples (up to 10 pooled larvae/colony) were taken from 23 colonies with spotty brood patterns in fall 2018 in order to detect infected larvae. Larvae were homogenized in phosphate-buffered saline (de Graaf et al. 2013). Aliquots of the homogenate were cultivated on MYPGP agar for *P. larvae* isolation, as previously described, and also diluted (1:1, v/v) in cetyltrimethylammonium bromide-buffer (CTAB) for direct molecular identification. DNA was extracted with phenol-chloroform-isoamyl-alcohol (PCI) thereafter. The pellet was frozen, resuspended in CTAB and reextracted with PCI (Evans et al. 2013). Bacterial colonies from the agar plates (MYPGP) as well as larval DNA-extracts were screened for *P. larvae* with specific primers (Govan et al. 1999) as described above.

### 16 S rRNA gene sequencing

At least one of the *P. larvae*-PCR-positive bacterial isolates per honey sample was further characterized by 16 S rDNA sequencing. Bacterial DNA was isolated using the QIAamp genomic DNA isolation mini kit for gram-positive bacteria (Qiagen GmbH, Germany). Each DNA extract was tested for the presence of *P. larvae* specific 16 S rDNA via PCR (Govan et al. 1999), and purified DNA was stored at −20 °C. Unambiguous species identification was carried out by bi-directional Sanger sequencing (Sanger et al. 1977) of the purified 973 bp PCR fragment with a Genetic Analyzer DNA Sequencer (Eurofins Genomics, Ebersberg, Germany).

Partial 16 S rRNA gene sequences of the isolates were compared to 16 S rRNA gene sequences available by the BLAST search (Altschul et al. 1990) in the National Centre for Biotechnology Information (NCBI) database (http://www.ncbi.nlm.nih.gov/). The genome sequences were deposited in NCBI GenBank under accession numbers OP437725 - OP437739. Multiple sequence alignments were performed using MultAlin (Corpet 1988).

### Genotyping of *Paenibacillus larvae*

The clonal bacterial QIAamp-kit generated genomic DNA isolations were also used for the differentiation of *P. larvae*. PCR amplification of repetitive elements (Versalovic et al. 1994) using enterobacterial repetitive intergenic consensus (ERIC-) sequences was performed, allowing the identification of five genotypes, ERIC I-V (Genersch et al. 2006; Beims et al. 2020). Bacterial isolates from fourteen honey bee colonies containing *P. larvae* spores were randomly selected from all apiaries for genotyping. PCR reactions were carried out according the procedure described in (Genersch et al. 2006). 10 µl of the PCR products were separated in standard 0.8% agarose gel, stained with Midori Green (Nippon Genetics, Japan), visualized by UV light, size-determined with 100-bp molecular size marker (Promega, USA) and photographed with a gel doc digital image capture system (Bio-Rad, Munich, Germany).

### Statistical analysis

Statistics were performed using R 4.2.1. [R Core Team, 2022](R-Development-Core-Team 2007). Significance levels were set to *p* ≤ 0.05 for all statistical tests, and p-values were adjusted for multiple testing using Bonferroni-Holm-correction (Holm 1979). The proportion of open brood cells within areas of capped brood cells in spore-free and spore-containg colonies were compared by Mann-Whitney U Test. Brood production was indicated by the ratio of capped brood cells with adult bee numbers, analyzed by linear regression (R-Package RcmdrPlugin. Kmggplot2) and compared between infected and uninfected colonies via Student’s t-test. Colonies without brood or < 600 bees were not included in the former analyses. Mixed effect models (LMM) were implemented using the lmer function within the lme4 R package (Bates et al. 2015). Post-hoc tests for differences in prevalence of AFB between seasons were performed with Tukey tests using the command ‘glht’(package’multcomp’). Differences in the number of colony-forming units per honey sample were compared using Student’s t-test. The effect of treatment with antibiotics on the prevalence of *P. larvae* and colony survival was tested using χ^2^. Graphs were produced using the R package ggplot2 (Wickham 2016). The spatial data included in the map (Fig. 1) were obtained from Geomolg, a geospatial information portal administered by the Palestine Ministry of Local Government. The data is publicly accessible and locally available free of charge. The map primarily includes historical Palestine along with its neighboring countries, the governorates in the West Bank, and apiaries. The locations of the apiaries were collected from the field using a Garmin GPS eTrex10, with geographical coordinates projected onto the map. ArcGIS Pro 3.1.5 was used to produce the map. The full data resource is available via the link: http://geomolg.ps.

## Results

### Prevalence of *Paenibacillus larvae*

During the two year survey (2017–2018), clinical symptoms of AFB were not detected. But spores of *P. larvae* were detected in honey samples of seven out of eight apiaries (87.5%), 22.3% (29/130) of the inspected colonies contained spores and 62.1% (18/29) of the spore-containing colonies died during the inspection period (Table 1). Colonies of two apiaries in Al-Dahiriya and Ubeidiya had high prevalences (59% and 53%), respectively (Table 1; Fig. 1). *P. larvae* was also detected in two of 23 larval samples (colony U37 and U40) taken from colonies that had a spotty brood pattern in fall 2018 and reflected the presence of spores in honey. The detection of the pathogenic agent in larvae indicated the outbreak of AFB in these colonies.

**Table 1:** Prevalence of *P. larvae* in honey bee colonies of Palestine

Apiary	Region	# of inspected colonies^1^	# of spore-containing colonies	# of dead spore-containing colonies	% of spore-containing colonies^3^	AFB-Treatment with oxytetracycline²
Bayt-Ula	Hebron	21	0	0	00.0	Yes
Majd-Dura	Hebron	17	1	1	5.9	Yes
Al-Dahiriya	Hebron	22	13	9	59.1	Yes
Battir	Bethlehem	22	1	0	4.5	Yes
Wadi-Fukin	Bethlehem	11	1	0	9.1	Yes
Hindaza	Bethlehem	10	2	0	20.0	No
Ubeidiya	Bethlehem	17	9	8	52.9	No
Shawawreh	Bethlehem	10	2	0	20.0	No
Sum		**130**	**29**	**18**		

### Regional and seasonal occurrence of *P. larvae*

**Fig. 2:**
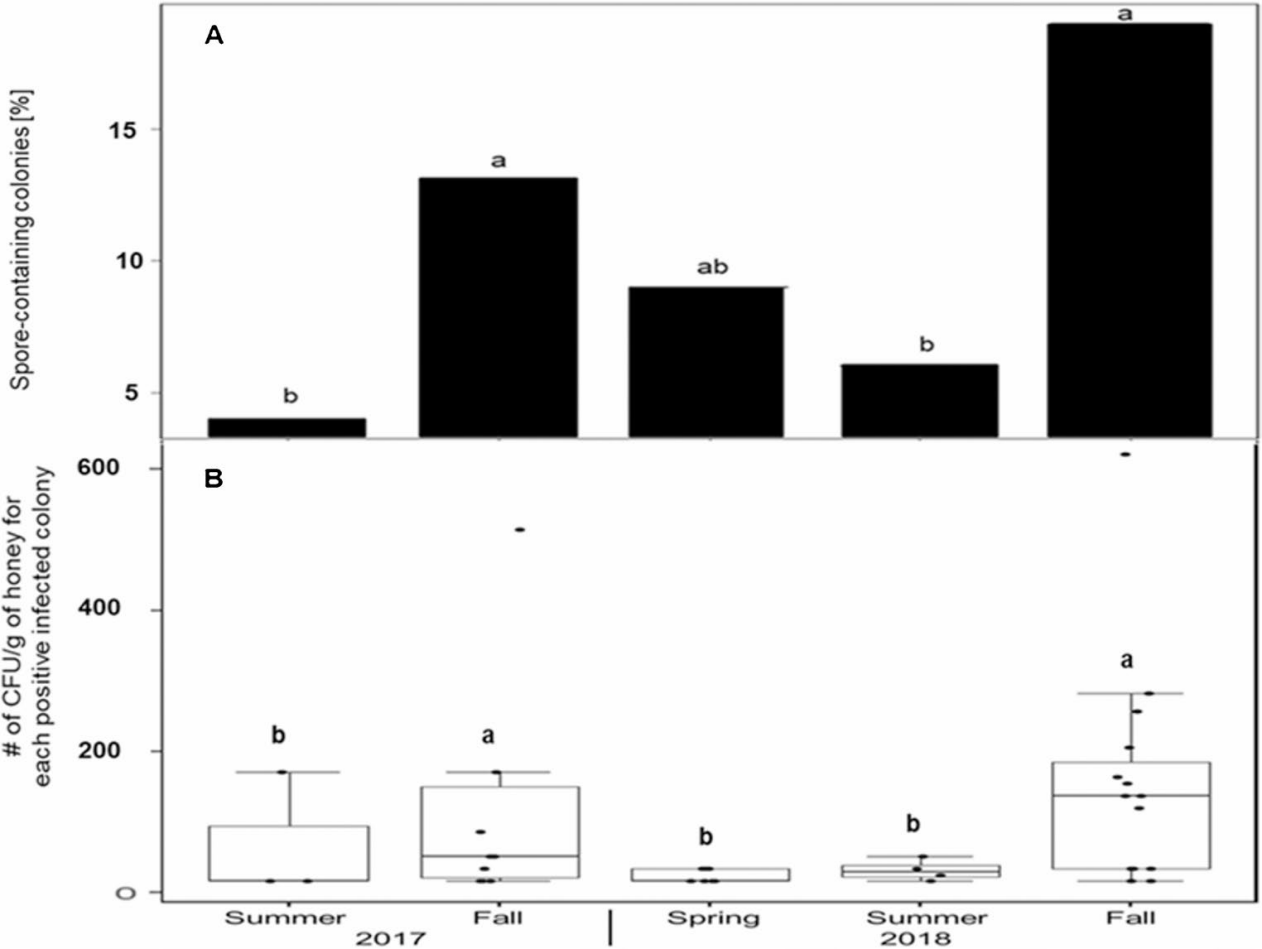
Seasonal variation of *P. larvae* prevalence **A** and spore number **B** in honey of Palestinian honey bee colonies (*n* = 80/season). (A) The number of spore-containing colonies was higher in fall than in the preceding summer (Tukey post-hoc test), (B) Spore number in spore-containing colonies (*n* = 3–15 colonies; CFU, median, max, min) peaked in fall (t-test). Lowercase letters denote significant differences (*p* < 0.05)

The occurrence of *P. larvae* did not differ between the two regions of West Bank (χ^2^ = 0,047, df = 1, *p* = 0.829; Table 1). However, the prevalence of spores varied between seasons (LMM; χ^2^ = 22.29, df = 2, *p* = 0.001; Fig. 2A). Most colony infections were found in fall (2017: *n* = 10, 2018: *n* = 15), whereas infection levels were significantly lower during preceding summer seasons (Tukey post-hoc test: *p* < 0.05, cf. supplementary Table S1). This seasonal pattern was reflected by the number of colony-forming units (CFU) in the honey samples (Fig. 2B). The highest CFU were found in fall 2017 and fall 2018. The latter was significantly higher than in the spring and summer of 2018 (t-Test, *p* = 0.009 resp. 0.013).

## Indicators for American foulbrood: open brood cells and brood production

**Fig. 3:**
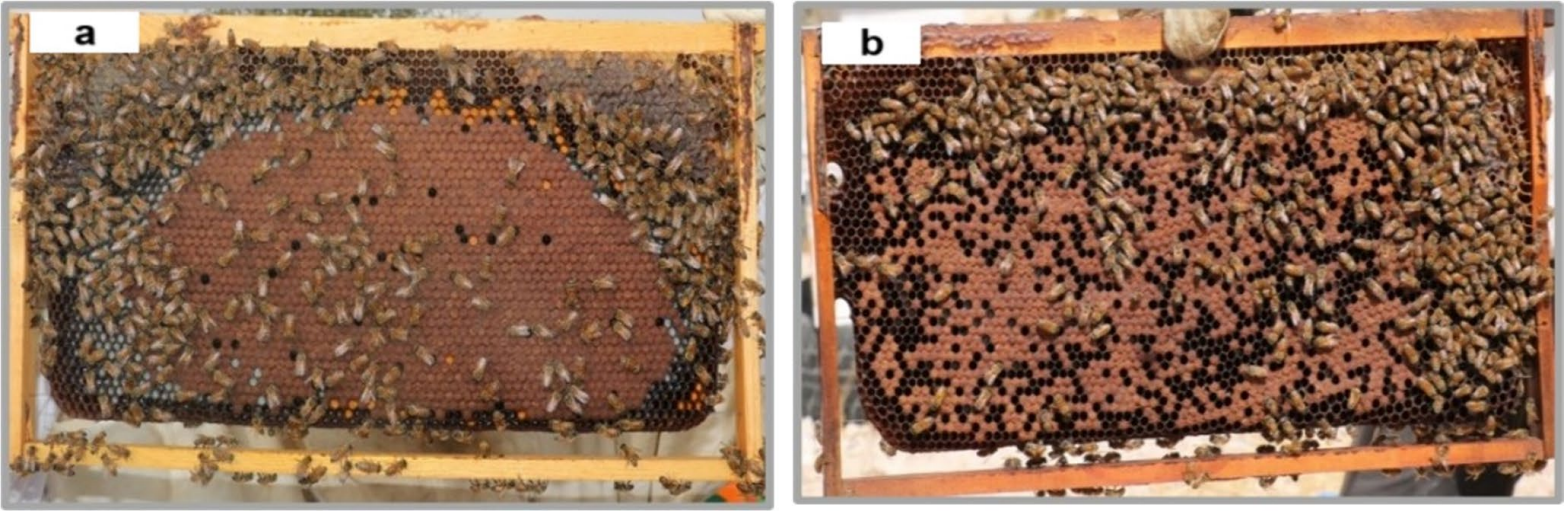
Pattern of capped brood cells in colonies without *P. larvae* spores detected **a **and in *P. larvae* spore containing colonies** b**. The photos are examples from Shawawra apiary (a) and from the Ubedyia apiary (b) respectively

**Fig. 4:**
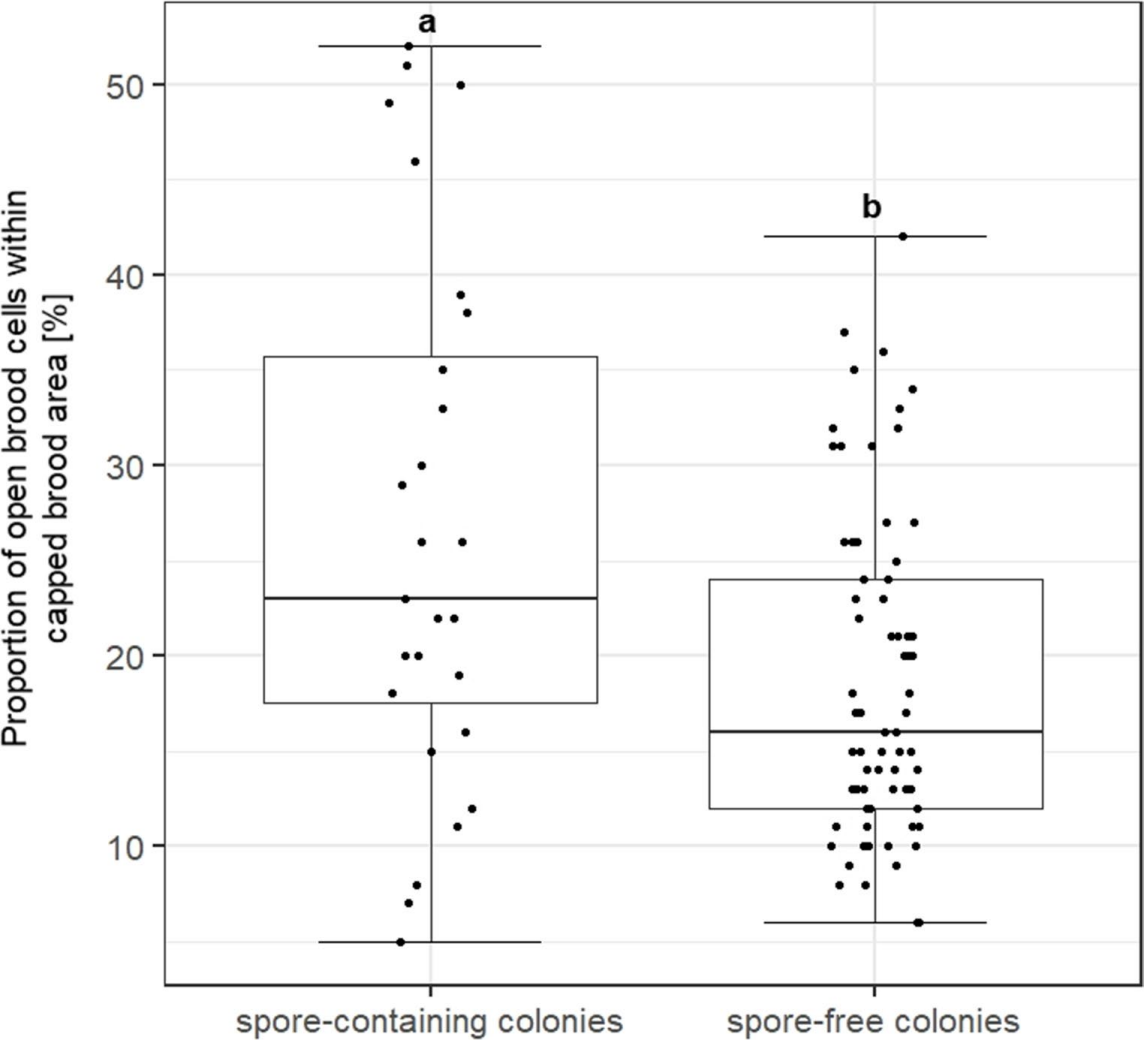
Proportion of open brood cells within areas of capped brood cells in spore-containing colonies (*n* = 29) and spore-free colonies (*n* = 71). Areas of $\:\ge\:$300 brood cells/colony were analysed. The box plots contain 1. and 3. quartiles split by the median, minimum and maximum. Differences between the two groups are significant (Mann-Whitney U-test, *p* = 0.0025)

Specific visual clinical symptoms as direct indicators for AFB, such as ropy larval remains, dark brittle scales of dried remain*s* firmly adhering to the brood cell and dead pupal tongues projecting from the remains were not detected during the inspections. Therefore, we analysed the parameters open brood cells within areas of capped brood cells (Fig. 3) and brood production/adult bee as indirect indicators for AFB. Both measures indicated failures in normal brood development. Spore-containing colonies had significantly higher proportions of open cells in areas of capped brood cells (median 23%) than spore-free colonies (median 16%) (Mann-Whitney U-test *p* = 0.0025; Fig. 4).

The brood production as indicated by the ratio of capped brood cell number and number of adult bees showed linear relations (supplementary Fig. S1) and differed between spore-free and spore-containing colonies (Student’s t-test, *p* = 0.020). Bees in spore-free colonies reared three times more brood/bee than bees in spore-containing colonies. Thus, both parameters, brood production/bee and proportion of open cells within areas of capped brood in relation total number of capped brood cells proved to be helpful, albeit indirect indicators for the presence of *P. larvae* in the absence of clinical AFB symptoms.

### Genetic identification and differentiation of *P. larvae*

All isolates from MYPGP-agar plates of the 29 spore-containing colonies were identified as *P. larvae* via PCR with primer pairs of 16 S rDNA expressing the expected fragment lengths of 973 bp (Govan et al. 1999) (Table 2). Many of the PCR fragments were sequenced (Table 2, GenBank OP437725 to OP437739), and all sequences match with known 16 S rDNA sequences from type strains of *P. larvae* (DQ079623.1 (Genersch et al. 2006), and FJ649364.1 (Hamdi et al. 2013) and fully sequenced *P. larvae* strains ERIC I (CP019651.1) and ERIC II (CP019652.1) (Beims et al. 2020).

**Table 2:** List of *P. larvae* isolates, molecular identification and honey bee colony survival status

#	Colony	Year of sample collection	Apiary	Region	AFB Identification		Strain	Status of colony
					PCR reaction	GenBank #	ERIC	
1	I2	2017	Al-Dahiriya	Hebron	+			died
2	I3N	2018	Al-Dahiriya	Hebron	+	OP437732		died
3	I5N	2018	Al-Dahiriya	Hebron	+			survived
4	I6N	2018	Al-Dahiriya	Hebron	+	OP437728	II	died
5	I7	2017	Al-Dahiriya	Hebron	+			died
6	I11	2017	Al-Dahiriya	Hebron	+			died
7	I12	2018	Al-Dahiriya	Hebron	+	OP437727	II	died
8	I13	2017	Al-Dahiriya	Hebron	+			died
9	I13N	2018	Al-Dahiriya	Hebron	+	OP437733	I	survived
10	I14	2018	Al-Dahiriya	Hebron	+			died
11	I15	2018	Al-Dahiriya	Hebron	+	OP437734	II	survived
12	I18	2018	Al-Dahiriya	Hebron	+	OP437725	I	survived
13	I19	2018	Al-Dahiriya	Hebron	+			died
14	U31	2018	Ubeidiya	Bethlehem	+		I	died
15	U34	2017	Ubeidiya	Bethlehem	+			died
16	U37	2017	Ubeidiya	Bethlehem	+			survived
17	U38	2018	Ubeidiya	Bethlehem	+	OP437736	I	died
18	U39	2018	Ubeidiya	Bethlehem	+	OP437737	I	died
19	U40	2017	Ubeidiya	Bethlehem	+			died
20	U41N	2018	Ubeidiya	Bethlehem	+	OP437738		died
21	U43	2018	Ubeidiya	Bethlehem	+	OP437739		died
22	U47	2018	Ubeidiya	Bethlehem	+			died
23	S4	2017	Shawawreh	Bethlehem	+		I	survived
24	S9	2018	Shawawreh	Bethlehem	+	OP437730	I	survived
25	E23	2017	Hindaza	Bethlehem	+		I	survived
26	E26	2017	Hindaza	Bethlehem	+			survived
27	H8	2017	Majd-Dura	Hebron	+		I	died
28	G3	2017	Wadi-Fukin	Bethlehem	+		I	survived
29	D23	2018	Battir	Bethlehem	+	OP437731	II	survived

Using ERIC primers, the bacterial isolates were further differentiated. Most *P. larvae* isolates represent the slow-killing ERIC-I-type, present in the apiaries of Ubeidiya, Hindaza, Majd, Wadi Fukin and Shawawreh. In Battir we found ERIC II, and in Al-Dahiriya we found both, ERIC I and II (Table 2).

### Effect of treatment with antibiotic oxytetracycline (OTC) against *P. larvae* on prevalence and colony survival

Five beekeepers regularly treated their colonies with the antibiotic OTC in the spring season but treatment did not affect the survival of the spore-containing colonies in both groups. Ten out of sixteen spore-containing colonies died in treatment colonies; eight out of thirteen spore-containing colonies died in untreated colonies, (χ^2^ = 0.058, df = 1, p-value = 0.98, cf. Table 1). Moreover, the presence of *P. larvae* in October was not dependent on the treatment with antibiotics during early spring – neither the number of spore-containing colonies (eight spore-containing of thirty untreated vs. seven spore-containing of fifty treated, (χ2 = 1.32, df = 1, *p* = 0.25; Supplementary Table 2) nor the number of CFU differed significantly between both subgroups (CFU_untreated colonies_ 102 ± 72, *n* = 8 and CFU_treated colonies_, 112 ± 34 CFU, *n* = 7, mean ± SE, t-test, *p* = 0.83; Supplementary Table 2).

## Discussion

This study reported the first record of *P. larvae* in Palestinian honey bee colonies. Unexpectedly, spores of *P. larvae* were widespread and occurred in seven of eight apiaries. At the colony level, 22.3% of the inspected colonies contained *P. larvae* spores and two *P. larvae* genotypes, the common ERIC I and the more virulent, fast killing ERIC II were identified. Moreover, 62.1% of the spore-containing colonies were presumably lost by AFB infection during the two-year study. This assumption is based on three facts: adult bee and brood analyses of the colonies using the Liebefeld method (Delaplane et al. 2013) showed that adult bees in spore-free colonies reared three times more brood/bee than bees in spore-containing colonies (Supplementary Fig. S1), spore-containing colonies had higher proportions of open brood cells within capped brood cell areas (Fig. 4) indicating failures in brood development and the colonies did not die due to food shortage since the dead colonies had food reserves of either honey or winter feed of more than 1 kg. However, it cannot be ruled out that other factors such as *Varroa destructor* and viruses might also influence colony mortality (vanEngelsdorp and Meixner 2010; Smith et al. 2013).

The high prevalence of *P. larvae* in Palestine from randomly selected colonies resembles the result of a previous study in Jordan (32.4%; Arabiat et al. 2012) and could be explained by several reasons. First, the lack of knowledge about the presence of the disease and the missing experience of beekeepers to recognize it. This was probably due to the absence of specific visual clinical symptoms and due to the removal the diseased larvae by hygienic bees as indicated by the spotty brood nest appearance (Genersch 2010). Moreover, the preventive application of antibiotics like OTC against AFB inhibits the growth of vegetative forms of *P. larvae* but do not kill the spores (Stephan et al. 2019) which still allows the spread of spores between colonies. In contrast to the high prevalence of *P. larvae* in Palestine, colony prevalences between 0.3 and 2% derived from honey samples were reported in most European countries, such as in Germany (Genersch et al. 2010), as well as in Uruguay (Antúnez et al. 2012). Similar low colony prevalences based on other sources such as adult honey bees occurred in New Zealand (0,47%) (Hall et al. 2021) The low prevalence of AFB is the direct consequence of systematic national monitoring approaches combined with effective eradication programs in these countries (Antúnez et al. 2012).

The results also show higher spore loads and prevalences in fall than in summer and spring. This seasonal pattern was similar to that in Slovenia (Žugelj et al. 2021) but different from most other studies which showed a lower prevalence of AFB in fall (EPILOBEE Consortium 2016 ( ; Morawetz et al. 2019). This higher prevalence in fall could be explained by the feeding of contaminated imported honey by beekeepers during summer nectar dearth and may also be affected by the treatment of 62,5% of all inspected colonies with the antibiotic OTC in spring. Its inhibitory effect on the vegetative forms of the bacteria did not persist since prevalence, colony survival and number of CFU did not differ statistically between OTC-treated and untreated colonies in fall. Antibiotic treatment against *P. larvae* is recommended by but were not realized by Palestinian beekeepers.

Typically, AFB disease is diagnosed based on presence of typical AFB symptoms and the identification of *P. larvae* agent (OIE, 2018; de Graaf et al. 2013). In this study, we looked for any clinical symptoms such as ropiness and scales during each inspection as well as identified the pathogen agent in the honey stored close to the brood nest, which was considered a proper measure of the colony infected by AFB disease when the clinical symptoms were absent (Forsgren and Laugen 2014; Bassi et al. 2018). Basically, *P. larvae* is identified by morphological characteristics, biochemical reactions, microscopic analysis, and molecular techniques (de Graaf et al. 2013). Also, in the current study, PCR techniques and DNA sequencing were applied to identify *P. larvae* using primers for 16 S rRNA gene (Govan et al. 1999). The sequences deposited in the NCBI database matched with 100% identity with sequences from Tunisia (FJ649358; (Hamdi et al. 2013), Italy (AY030079; (Lauro et al. 2003), Germany (DQ079622 and CP019651.1; (Genersch et al. 2006; Beims et al. 2020) and USA (CP019687 representing the reference strain ATCC-9545; (Dingman 2017) and with 99% with an isolate from the neighbouring country Saudi Arabia (KR780760; (Ansari et al. 2017).

Specifically, the different virulences of ERIC genotypes of *P. larvae* had driven us to determine these genotypes in our isolates. The genotypes ERIC I and ERIC II occurred in six and two apiaries respectively. ERIC I seems to be more prevalent *P. larvae* genotype worldwide (Loncaric et al. 2009; Rusenova et al. 2013; Morrissey et al. 2015; Chemurot et al. 2016; Krongdang et al. 2017; Bassi et al. 2018; Hristov et al. 2021). So far, ERIC II has been identified in some countries (Morrissey et al. 2015; Hirai et al. 2016; Hristov et al. 2021). Recently, in Slovenia and Czech republic ERIC II was found more predominant than ERIC I (Biová et al. 2021; Žugelj et al. 2021). Both genotypes were isolated in one apiary (Aldahryia), showing that mixed infections of the ERIC I and II could occur as already reported (Loncaric et al. 2009; Bassi et al. 2015; Ågren et al. 2017). Notably, ERIC I-infected colonies often exhibit more recognizable and prolonged symptoms, such as the classic ropy larval remains, which increases the likelihood of detection during inspections (Beims et al. 2020; Hristov et al. 2021). This leads to a potential detection bias, making ERIC I appear more prevalent than it may actually be. In contrast, ERIC II tends to cause faster larval death and colony collapse, possibly resulting in underdiagnosis (Genersch et al. 2005). Therefore, our genotype frequency results may reflect the less easy detection of ERIC II and not necessarily the real genotype frequencies since hygienic workers remove infected larvae from uncapped cells (Brødsgaard et al. 2000) in medium fast killing ERIC II genotypes (Genersch et al. 2006; Beims et al. 2020) resulting in empty cells and leaving a scattered brood pattern as detected in Palestinian bee colonies and also resulting in presumably lower spore loads by ERIC II infections (Rauch et al. 2009).

Due to the discrepancy between the detected high prevalence of *P. larvae* spores and the absence of typical AFB symptoms (Genersch 2010) in Palestinian colonies, the number open brood cells within areas of the capped brood cells were used as the only remaining visual proxy for AFB. They were significantly higher in spore-containing than healthy colonies. However, this symptom provides only indirect evidence because it is also present with other brood diseases such as *Varroa* mite (Morawetz et al. 2019), European foulbrood disease (Forsgren 2010) and Sacbrood virus (Bailey and Fernando 1972). But spotty brood pattern and dwindling colony strength may serve as helpful initial indicators of AFB disease in Palestine for the beekeepers and require once detected further specific identification steps. Moreover, brood production was negatively correlated with presence of spores in colonies. Spore-free colonies reared three times more brood than spore-containing colonies. As a consequence the number of emerging bees should decrease, disrupting bee performance, and cause weakening colonies that finally die (Rauch et al. 2009; Stephan et al. 2020).

The origin of *P. larvae* in Palestine, its first occurrence and transmission route are unknown. In general, transmission routes include biological transmissions by robbing and drifting bees, and other vectors such as *Varroa* mite and small hive beetle, but also beekeeping activities such as feeding with imported contaminated honey and pollen, trading colonies or queens, exchanging frames between honey bee colonies. or transmission via contaminated beekeeping tools and clothes and migratory beekeeping (Jacobs 2002; Fries et al. 2006; Lindström et al. 2008b; Schäfer et al. 2010; Anjum et al. 2015; Jończyk-Matysiak et al. 2020). Since migratory beekeeping is limited in Palestine, transmission by foraging bee activities seems unlikely, but colonies in one hot spot (Al-Dahiriya apiary) were fed with imported honey during summer nectar dearth which might explain the co-appearance of ERIC I and II within this apiary, whereas colony trading may explain the infections in Ubedyia apiary and Battir. Such a transmission route was reported from Sweden where one beekeeper who sold infected colonies was the main source for AFB transmission between apiaries (Ågren et al. 2017). However, our sampling and monitoring approach only allow indirect conclusions. Deciphering the transmission routes would require to use molecular identification tools such as multilocus sequencing techniques (Morrissey et al. 2015), multiplex PCR (Okamoto et al. 2022) and/or highly sensitive quantitative PCR (Papić et al. 2024) in the future, a tightly scheduled sampling scheme and the extension of sampling to adult honey bees (Bassi et al. 2018) as well as to honey and bee colony trading in the future.

High numbers of *P.larvae*-spore-containing colonies and the loss of around 62% of the spore-containing colonies throughout the study, obliged us to be more aware of combating AFB in Palestine. Three major treatments are effective (Genersch 2010): Burning colonies and contaminated hive material, the less harsh shook swarm method and the use of antibiotics. However, the latter treatment method is banned in most European countries because it leads to unacceptable residues in honey. In addition, antibiotics only mask the symptoms of AFB rather than curing it (Pettis and Feldlaufer 2005; EPILOBEE Consortium et al. 2016; Krongdang et al. 2017).

Currently *P. larvae* infections in Palestine would result in an eradication of the infected colonies. Successful national strategies like in New Zealand where AFB is controlled by legislation and beekeepers are required to undertake regular hive inspections and destroy infected colonies within seven days of after being discovered and quarantine management lead to low prevalence of 0.085% (Hall et al. 2021). In Uruguay early detection by continuous monitoring and control programs that include the national authorities organizing extension activities resulted in a decrease of the AFB prevalence from 51% in 2001–2002 to only 2% in 2011 (Antúnez et al. 2012). Thus, a valuable and helpful strategy to control this disease in Palestine should include early detection by continuous colony monitoring, treatment regimens with shook swarm method or eradication of infected colonies, quarantine management, refrain from the use of antibiotics and beekeeper education programs.

## Conclusions

This study is the first to report on the prevalence of *P. larvae* in Palestinian honeybee colonies.The absence of typical clinical symptoms reveal significant gaps in current AFB surveillance methods. The widespread occurrence of the disease, coupled with a 62% loss of spore-containing colonies, underscores the urgent need for improved diagnostic strategies, surveillance protocols, and biosecurity practices. We recommend targeted training for beekeepers to recognize early infection signs and suggest that policymakers implement region-specific AFB management guidelines, including antibiotic stewardship and resistance monitoring. Given the unknown transmission routes within Palestine, further research is needed to trace the spread of *P. larvae*, particularly the more virulent ERIC II genotype, using molecular techniques. Collaborative efforts with neighboring countries could enhance these initiatives, helping to mitigate the impact of AFB and safeguard colony health in the region.

## Supplementary Information

Below is the link to the electronic supplementary material.

ESM 1 DOCX (55.6 KB)

## Data Availability

Datasets used can be required to corresponding author.
