# Supplementary material for: High prevalence of Paenibacillus larvae, the pathogenic agent of American foulbrood disease, in Palestinian honey bee colonies
Source: Vet Res Commun. 2026 Jan 19;50(2):111. doi: 10.1007/s11259-025-11034-x (PMC12816049; doi:10.1007/s11259-025-11034-x)
Supplement: Supplementary file 1 — DOCX (55.6 KB) [file 11259_2025_11034_MOESM1_ESM.docx]

Supplementary Information- Veterinary Research Communications

**High prevalence of *Paenibacillus larvae*, the pathogenic agent of American foulbrood disease, in Palestinian honey bee colonies**

Mohammad Alqurneh^1,^ ^2*^, Nino Tuan Phong Bergmann^1^, Islam Nairoukh^3^, Abdul-Jalil Hamdan^3^ and Hans-Hinrich Kaatz^1^

^1^ Institute of Biology, Dept. Zoology, Martin-Luther-University Halle-Wittenberg, Germany

^2^ Department of pest monitoring and control, General directorate of plant protection and inspection services, Ministry of Agriculture, Ramallah, Palestine

^3^ Department of Plant Protection and Production, Agriculture Science College, Hebron University, Hebron, Palestine

**Corresponding author**

Mohammad Alqurneh

[Mohammad.alqurneh@moa.pna.ps](mailto:Mohammad.alqurneh@moa.pna.ps)

**Supplementary Table 1. Seasonal differences in *P. larvae* prevalence.**

| **Seasonal comparison** | **estimate** | **Std. Error** | **z value** | **P** |
| --- | --- | --- | --- | --- |
| summer 2017 & autumn 2017 | 0.09507 | 0.03685 | 2.580 | **0.029667** |
| summer 2017 & spring 2018 | 0.06562 | 0.03791 | 1.731 | 0.139155 |
| summer 2017 & summer 2018 | 0.02131 | 0.0382 | 0.558 | 0.576955 |
| summer 2017 & autumn 2018 | 0.15001 | 0.0387 | 3.876 | **0.000796** |
| autumn 2017 & spring 2018 | 0.02945 | 0.03726 | 0.79 | 0.495295 |
| autumn 2017 & summer 2018 | 0.07377 | 0.03757 | 1.963 | 0.093026 |
| autumn 2017 & autumn 2018 | 0.05494 | 0.03811 | 1.442 | 0.224117 |
| spring 2018 & summer 2018 | 0.04431 | 0.03734 | 1.187 | 0.320979 |
| spring 2018 & autumn 2018 | 0.08439 | 0.03792 | 2.226 | 0.055796 |
| summer 2018 & autumn 2018 | 0.12871 | 0.03697 | 3.481 | **0.002498** |

The statistical analysis (LMM, Tukey post-hoc test with Bonferroni corrections) was conducted to show seasonal differences in ***P. larvae*** prevalence.

**Supplementary Figure 1.** Brood production in spore-containing and spore-free colonies

(a) (b)

**spore-containing**

**colonies**

**spore-free colonies**

**Supplementary Figure 1.** Brood production as indicated by the relation of the number of capped brood cells to the number of adult bees in spore-free (a) and spore-containing colonies (b). Data on the two apiaries with highest AFB prevalence (Al-Dahiriya and Ubeidiya) were analyzed. Linear regression and 95% confidence interval. Bees in spore-free colonies produce about three times more brood (y= 1.906x - 651.2, r=0.88, p=0.0006) than those in spore-containing colonies (y = 0.6754x + 2326, r=0.29,p=0.027). The ratio of capped brood cells to adult bee numbers is significantly different between spore-containing (n=17) and spore-free (n=8) colonies (t-test, p=0.0195).

**Supplementary Table 2.** Colony-forming units (CFU) of *Paenibacillus larvae* spores detected in oxytetracycline treated or untreated honey bee colonies in October 2018

| **Treatment with oxytetracycline*** | | | **Untreatment with oxytetracycline** | | |
| --- | --- | --- | --- | --- | --- |
| **Colony** | **Apiary** | **CFU/g of honey** | **Colony** | **Apiary** | **CFU/g of honey** |
| I3N | Al-Dahiriya | 34 | U31 | Ubeidiya | 137 |
| I5N | Al-Dahiriya | 120 | U38 | Ubeidiya | 621 |
| I13N | Al-Dahiriya | 290 | U39 | Ubeidiya | 17 |
| I14 | Al-Dahiriya | 17 | U40 | Ubeidiya | 17 |
| I15 | Al-Dahiriya | 137 | U41N | Ubeidiya | 34 |
| I18 | Al-Dahiriya | 206 | U43 | Ubeidiya | 257 |
| I19 | Al-Dahiriya | 164 | U47 | Shawawreh | 34 |
| D23 | Battir | 154 |  |  |  |

* Oxytetracycline treatment was performed once in spring 2018.
